# Multi-tissue Siberian sturgeon RNA sequencing data

**DOI:** 10.1016/j.dib.2020.105820

**Published:** 2020-06-07

**Authors:** Christophe Klopp, Cédric Cabau, André Lasalle, Santiago Di Landro, Gonzalo Greif, Denise Vizziano-Cantonnet

**Affiliations:** a INRAE, SIGENAE, MIAT UR875, F-31326, Castanet Tolosan, France, ‘SIGENAE, GenPhySE, Université de Toulouse, INRAE, ENVT, F-31326, Castanet Tolosan, France; b Laboratorio de Fisiología de la Reproducción y Ecología de Peces, Instituto de Biología, Facultad de Ciencias, Universidad de la República Oriental del Uruguay, Iguá 4225, Montevideo, 11400, Uruguay; c Laboratorio de Interacción Hospedero-Patógeno/Unidad de Biología Molecular, Instituto Pasteur de Montevideo, Uruguay

**Keywords:** Siberian sturgeon, Transcriptomics, Brain-pituitary-gonad axis, Anterior-kidney, Kidney, Stomach, Liver, Embryos

## Abstract

Siberian sturgeon, Acipenser baerii, is a commercially valuable fish for flesh and caviar production and a threatened species. We produced transcriptomic data for ten tissues with relevance to puberty, reproduction, early development, growth and food intake. The data includes RNA-Seq read sets of brain, pituitary, anterior-kidney, kidney, stomach, liver, heart, embryonic, pre-larval, and immature gonad sequences. Tissues were collected from sex differentiated fish (17 to 42 months of age, 66 to 85 cm) RNA was extracted and sequenced. Our purpose is to facilitate fundamental studies of sturgeon physiology to wild and aquaculture populations management.

Specifications Table

**Table:** 

Subject	Biology
Specific subject area	Transcriptomics, physiology and aquaculture
Type of data	Raw RNA-Seq data and assembled reference transcriptome assembly
How data were acquired	Illumina MiSeq Illumina HiSeq 2500
Data format	Raw data and assembly information
Parameters for data collection	Tissues collected from immature fish: pituitary, anterior-kidney, kidney, stomach, liver, gonads Tissues collected from gametogenetic fish: brain Tissues collected from sex undifferentiated fish: embryonic, pre-larval
Description of data collection	Tissues sequenced in a pool: pituitary, testis, ovary, anterior kidney, kidney, heart, stomach, liver. Tissues sequenced individually: brain, pituitary, anterior kidney, kidney, heart, stomach, liver.
Data source location	Laboratorio de Fisiología de la Reproducción y Ecología de Peces, Facultad de Ciencias, Universidad de la República Oriental del Uruguay. Country: Uruguay Site of collection: Estuario del Plata sturgeon farm, San Gregorio de Polanco, Tacuarembó, Uruguay
Data accessibility	Raw data of RNA Seq analysis are available on Sequence Read Archive (SRA) database and connected with BioProject PRJNA589958,https://www.ncbi.nlm.nih.gov/bioproject/PRJNA589958 Reference transcriptome assembly is available at the NCBI in TSA GICB00000000https://dataview.ncbi.nlm.nih.gov/object/PRJNA589958?reviewer=ik54sg8njqstt0j9psepieaona

## Value of data

• The dataset will facilitate research on topics of interest in Siberian sturgeon aquaculture such as puberty, reproduction, growth, food intake, and immunology. Insights into these processes will improve management of both wild and aquaculture populations.
• This data benefits the community of scientists working on fish biology and aquaculture. It also can be used for fish evolution studies.
• This data includes different tissues enabling to better understand different functions. For example, the data on the brain-pituitary axis will be helpful for studies on puberty, growth and food intake control.
• This is the first Siberian sturgeon RNA-seq multi-tissue data set.

## 1 Data

The Siberian sturgeon, Acipenser baerii, is a non-teleost ray-finned fish (Actinopterygii) which face critical conservation problems [1,2] due to overfishing, incidental fishing, river pollution, dam construction, other environmental disruptions, and poor fishery management [1], [2], [3], [4]. There has been sharp decline in commercial Siberian sturgeon catches [5] and has now spread to 49 countries worldwide [6]. Knowledge of sturgeon physiology and genetics is less advanced than for other industrial species such as salmonids.

Here, we present a novel Siberian sturgeon multi-tissue data set including brain, pituitary, gonadal, liver, stomach, kidney, anterior kidney, heart, embryonic, and pre-larval transcriptomes, with the goal of facilitating crucial research on topics of interest in sturgeon physiology, such as puberty, reproduction, growth, food intake, and in immunology. Insights into these processes will improve management of both wild and aquaculture populations.

Samples of brain, pituitary, gonads, liver, stomach, kidney, anterior kidney were taken from males and females aged 15–42 months (Table 1). Samples were also collected from embryos one day prior to hatching and pre-larvae on the day of hatching.

**Table 1:** Characteristics of fish used to obtain tissue samples for individual and pool sequencing.

Fish Id	Age	Sex	Stage	Tissue samples	Total length/cm	Total weight/kg	Individual (I) and/or Pool (P)
259	17	Male	Immature	Testis	77.5	1.8	P
262	17	Female	Immature	Ovary	75.5	1.6	P
380	15	Male	Non-reproductive	Brain	66.8	0.879	I
381	15	Female	Non-reproductive	Anterior-kidney	66.1	0.851	I, P
381	15	Female	Non-reproductive	Kidney	66.1	0.851	I, P
381	15	Female	Non-reproductive	Heart	66.1	0.851	I, P
382	15	Male	Non-reproductive	Stomach	64.1	0.842	I, P
384	20	Male	Non-reproductive	Liver	76.5	1.85	I, P
630	42	Male	Non-reproductive	Pituitary	85.5	2.44	I, P

In a first experiment a pool of tissues was sequenced using Illumina MiSeq (Table 2) and in a second experiment tissues were sequenced individually (Table 2). Raw data correspond to Fastq for RNA-Seq reads and fasta for assembled contigs.

**Table 2:** Sample identification (Id), library name, SRA and SAMN, sequencer and quality of sequences produced.

Fish Id	Tissue samples	Library name	SRA files	SAMN files	Sequencer	Nb reads (1)	Alignment rate (2)	Q20 ratio (3)
380	Brain	380C	SRR10466940	SAMN13295011	Illumina HiSeq 2500	92,338,008	95.10	99.28
381	Anterior-kidney	381Int	SRR10466939	SAMN13295012	Illumina HiSeq 2500	71,233,652	97.11	99.36
381	Kidney	381Rein	SRR10466938	SAMN13295013	Illumina HiSeq 2500	62,440,734	96.56	99.39
381	Heart	381Coeur	SRR10466937	SAMN13295014	Illumina HiSeq 2500	69,179,714	97.30	99.37
382	Stomach	382Est	SRR10466936	SAMN13295015	Illumina HiSeq 2500	66,452,854	98.67	99.33
384	Liver	384Foie	SRR10466935	SAMN13295016	Illumina HiSeq 2500	70,511,690	98.67	99.41
630	Pituitary	630Hyp	SRR10466934	SAMN13295017	Illumina HiSeq 2500	69,219,726	94.81	99.4
Nd	Embryos.One day prior hatching	Sexsturg-E1-E2	SRR10466932	SAMN13295019	Illumina HiSeq 2500	80,075,510	97.04	97.43
Nd	Pre-larvaeDay of hatching	Sexsturg-L1-L5	SRR10466931	SAMN13295020	Illumina HiSeq 2500	85,793,960	96.78	97.12
259, 262, 381, 382, 384, 630	Pool of tissues, testis, ovary, anterior kidney, kidney, heart, stomach, liver, pituitary	poolTejidosEsturion	SRR10466933	SAMN13295018	Illumina MiSeq	17,594,907	99.08	97.16

(1) Number of reads

(2) Alignment rate: is the number of sequences aligned on the de novo transcriptome reference divided by the total number of sequences of the sample expressed in percent

(3) Q20 ratio is the number of raw read base pairs having a quality score equal or over 20 divided by the total number of read base pairs of the sample expressed in percent.

## 2 Experimental design, materials, and methods

### 2.1 Ethics statement

Research procedures involving animal experimentation complied with international principles on the use and care of laboratory animals and Uruguayan regulations on animal welfare. The protocol was approved by the Comisión de Etica en el Uso de Animales of the Comisión Honoraria de Experimentación Animal CHEA of Uruguay (Authorization Number 240,011–002,227–16).

### 2.2 Experimental animals and rearing procedures

Siberian sturgeon individuals were obtained from a fish farm (Estuario del Plata, Uruguay) and reared at natural conditions [7]. Usinga batch of embryos arrived from Poland to Uruguay and cultured at the Estuario del Plata farm (San Gregorio de Polanco, Tacuarembó), we collected (embryos one day prior to hatching and pre-larvae on the day of hatching). For fish aged from 15 to 42 months, we also used fish cultured at Estuario del Plata that came at embryo stage from Poland. They were sacrificed by spinal transection to obtain brain, pituitary, gonads, liver, stomach, kidney, anterior kidney (Table 1).

### 2.3 RNA extraction, cDNA library construction, and illumina sequencing

RNA from various tissues (pituitary, testicular, ovarian, liver, stomach, kidney, anterior kidney, heart, Table 1) was extracted using the Illustra RNAspin Mini RNA Isolation Kit (GE Healthcare) according to manufacturer instructions, and quality was assessed using the Agilent 2100 Bioanalyzer. cDNA synthesis was carried out on 4 μg of total RNA. The RNA samples conformed to the required purity criteria (A260/A230 and A260/A280 >1.8) and quality levels (RIN >8) for cDNA library preparations for sequencing. 0.5 μg of RNA of each tissue were mixed to create the pool. Sequencing was performed using the Epicenter kit (ScriptSeq™ v2 RNA-Seq Library Preparation Kit) on an Illumina MiSeq system with paired-end read length of 2 × 75 base pairs at the Unidad de Biología Molecular of Institut Pasteur in Montevideo, Uruguay.

Individual samples of brain, pituitary, liver, stomach, kidney, anterior kidney, embryonic, and pre-larval tissues were sequenced to provide deeper coverage. For the individual samples, total RNA was extracted and libraries constructed on a Tecan EVO200 liquid handler using the Illumina TruSeq Stranded mRNA sample prep kit. Libraries quality were checked on an Agilent High Sensitivity DNA Kit and quantified with the KAPA Library Quantification Kit to ensure accuracy and performed on an Illumina HiSeq 2500 system (high-throughput mode) using a paired-end read length of 2 × 100 base pairs with the Illumina TruSeq SBS Kit, v3. Individual tissue samples were sequenced at the Plateforme Génomique (INRA Auzeville in Castanet-Tolosan, France). Assembly and annotation were performed using both the pooled and individual tissue data. The number of reads per set ranged between 17,594,907 and 92,338,008. The read quality was assessed by calculating a Q20 ration corresponding to the fraction of nucleotides having a quality score over 20 for all the read of each sample (Table 2). The Q20 ratio ranged from 97.12 to 99.41%.

### 2.4 Transcriptome assembly

The transcriptome was assembled in two steps using the de novo RNA-Seq Assembly Pipeline (DRAP) 1.9 [8]. First, 10 tissue assemblies were performed with runDrap using 20 million read-pairs for each sample but the pool for which all the reads where used. Second the resulting contigs were merged with runMeta to produce the final reference file.

The transcriptome quality was checked using BUSCO (version 3.0.0) [9] using the Actinopterygii reference protein set (actinopterygii_odb9). Over 90% (4147/4584) of the BUSCO expected proteins were found in unique or duplicated copies in the set. The read quality was also re-assessed using the read versus contig alignment rate (Table 2) ranging from 94.81 to 99.08%. The alignment was performed with bwa [https://doi.org/10.1093/bioinformatics/btp324] mem version 0.7.12-r1039 with default parameters and the alignment rate was calculated on the bam file produced with samtools view and flagstat [https://doi.org/10.1093/bioinformatics/btp352] version 1.3.1 using default parameters.

## Declaration of Competing Interest

The authors declare that they have no known competing financial interests or personal relationships that could have appeared to influence the work reported in this paper.

## Appendix Supplementary materials

Image, application 1
